# Comparative Study of Different Measurement Methods for Characterizing Rheological Properties of Lubrication Layer

**DOI:** 10.3390/molecules26133889

**Published:** 2021-06-25

**Authors:** Yu Liu, Rui Jing, Peiyu Yan

**Affiliations:** 1 Department of Civil Engineering, Tsinghua University, Beijing 100084, China; liuyu_ustb@163.com (Y.L.); michael_jing2016@yahoo.com (R.J.); 2 China Construction Electric Power Construction Co., Ltd., Shenzhen 518034, China

**Keywords:** lubrication layer, rheological measurement, tribometer, Sliper, mortar, pumping

## Abstract

The lubrication layer plays a governing role in predicting the pumpability of fresh concrete. To determine the effect of measurement methods on the characterization of the rheological properties of the lubrication layer, different measurement systems, including Sliper, tribometer, and the utilization of a mortar rheometer, were employed. The rheological properties and workability of bulk concrete were measured in parallel to investigate the correlation between them and the rheological properties of the lubrication layer. The results show that the measured values of the rheological parameters of the lubrication layer differ due to the systematic deviation between different measurement methods. The results obtained by both tribometer and mortar rheometer were well-correlated, having a linear relationship with the rheological parameters of bulk concrete. The correlation coefficient between results gained with Sliper and rheological parameters of concrete or lubrication layer determined with other methods was not high enough. Addition friction led to the large accidental error and overestimated yield stress obtained with Sliper. The workability of concrete is only suitable for characterizing the rheological properties of bulk concrete.

## 1. Introduction

Concrete pumping has become a common technology at present for pouring concrete during the construction of super high-rise buildings, mass concrete structures, and long-distance tunnels [1,2,3]. Concrete can be transported efficiently to the construction platform or formwork by pumping, which can speed the construction with low cost and high quality [4,5]. With the accumulation of construction experience and the improvement of pumping equipment [6,7], concrete pumping has been continuously improved to be a mainstream construction technology [8,9]. In order to ensure the smooth implementation of concrete pumping, it is necessary to evaluate the pumpability of concrete before pumping [10]. Concrete pumpability is a comprehensive index, which characterizes the ability of concrete to flow stably in a pipe under pressure. The workability of concrete is often used in real construction to evaluate its pumpability [11,12,13]. However, since the extensive utilization of highly flowable concrete (HFC) and self-compacting concrete (SCC), it is no longer reliable to estimate the pumpability of concrete only by testing its workability. At present, establishing the relationship between pumping pressure and flow rate of concrete is a straightforward and most accurate way to estimate its pumpability [14,15]. Therefore, the moving state of concrete in the pipe needs to be deeply understood.

Generally, when the fresh concrete is in a steady flow state through the pump pipe, a lubrication layer is formed near the pipe wall [16]. Depending on the applied shear stress and the yield stress of fresh concrete near the pipe wall, the concrete can exist in the form of lubrication layer and plug flow or lubrication layer, shearing flow, and plug flow during the pumping process [17,18]. Many studies have shown that the decisive factor in improving concrete pumpability is the lubrication layer formed between the pipe wall and the bulk concrete [19,20,21]. Pumping of concrete is impossible without the formation of a lubrication layer [22,23]. The shear-induced particle migration, the bleeding tendency of concrete, and the wall effect were considered the main reasons for the formation of a lubrication layer, which together led to a centripetal migration of coarse aggregate across the cross-section of the pipe [24,25,26]. Therefore, the lubrication layer is usually considered to be the constitutive mortar of fresh concrete. Some researches indicated that the yield stress and viscosity of the lubrication layer are small compared with that of bulk concrete, but the rheological properties of the lubrication layer directly determined the pressure loss along the pumping process [27]. Some authors have found that the thickness of the lubrication layer was about 2 mm through the measurement with ultrasonic velocity profiler, which may depend on the volume fraction of aggregate and the diameter of the pump pipe [28]. It was also pointed out that the thickness of the lubrication layer ranged from 1 mm to 9 mm because of the difference in concrete compositions, including the water-cement ratio and fine aggregate content [29]. Many experiments have been carried out to study the effects of different factors, such as aggregate, mineral admixtures, superplasticizers, and pump pipe diameter, on the rheological properties of lubrication layer and bulk concrete and their pumping performance [30,31,32,33,34,35]. Some pump pressure-flow rate models have been proposed based on the rheology and applied to the prediction of concrete pumpability [36,37]. However, the accuracy of the prediction depends on the measurement of basic rheological parameters of the lubrication layer. Different measuring results of rheological parameters may lead to contradictory predictions of concrete pumpability. Therefore, the utilization of an accurate and convenient measurement for determining the rheological properties of the lubrication layer is conducive to the evaluation of concrete pumpability.

There are some measurement methods available to measure the rheological parameters of the lubrication layer. First, a tribometer is often used to determine the rheological properties of the lubrication layer [38,39,40]. Its structure and measurement method are similar to that of a coaxial cylinder rheometer, but the surface of the inner cylinder in a tribometer is smooth. The concrete in the tribometer need not be regarded as homogeneous, which is different from that of a concrete rheometer. The concrete is sheared on the surface of the rotating inner cylinder, and particles migrate to form a thin mortar layer. This is similar to the formation of the lubrication layer in the pump pipe. Therefore, it can be utilized to determine the rheological properties of the lubrication layer. Different types of tribometers have been invented and applied to practical engineering [37,41]. Second, as mentioned above, it has been considered that the lubrication layer consists of the constitutive mortar of fresh concrete [24,28]. Hence, the rheological properties of the mortar wet-screened from the fresh concrete can be used to represent the rheological properties of the lubrication layer. Some investigations have demonstrated the effectiveness of this method [27]. Moreover, the Sliding Pipe Rheometer (Sliper) developed based on the principle of the Kaplan model is the latest device used to evaluate the concrete pumpability, which is convenient to carry and operate [42]. During testing of Sliper, the fresh concrete remained stationary, and the outer pipe slid by changing the counterweight to simulate the process of concrete pumping and produce a lubrication layer. This was closer to the actual formation of the lubrication layer in the pump pipe compared with the tribometer. A pressure sensor and distance sensor were used to measure the pressure and sliding velocity in the sliding process, respectively. Then, the relationship between pumping pressure and flow rate could be obtained. The intercept and slope of the applied linear equation corresponded to the yield stress and viscosity of the lubrication layer, respectively. Sliper has been successfully applied to the prediction of concrete pumpability in field tests [43,44].

In general, the three methods for measuring the rheological properties of the lubrication layer are feasible in principle. However, the pumpability prediction models require absolute values of the rheological parameters of the lubrication layer. Due to the low value of the viscosity and yield stress for the highly flowable concrete, it is sensitive to the type of measurement system. Slight errors in the measurement can result in an inaccurate pumpability prediction of the concrete. Few studies have evaluated the three measurement methods simultaneously. The difference and stability of the results obtained by different measurement systems have not been fully understood, which is important to guide the determination of the rheological properties of the lubrication layer in actual engineering.

The present study was designed to investigate the effects of different measurement systems on the rheological properties of the lubrication layer. The tribometer and Sliper test were employed to determine the yield stress and viscosity of the lubrication layer. The mortar wet-screened from the fresh concrete was also measured with a rheometer to acquire the yield stress and viscosity of the lubrication layer. Simultaneously, the workability and rheological properties of fresh concrete were investigated and compared with the lubrication layer measured using the three methods.

## 2. Experimental

### 2.1. Materials

P∙I 42.5 Portland cement, which conforms to Chinese National Standard GB 175-2007 (equivalent to European CEM I 42.5 [45]), was used. Fly ash and silica fume conforming to Chinese National Standard GB/T 1596 and GB/T 27690, respectively, were also utilized for the preparation of the concrete used in this study. The chemical composition of the raw materials is shown in Table 1. The crushed limestone between 5 and 10 mm and natural river sand with fineness modulus of 2.6 was utilized as coarse and fine aggregates. The density of the coarse and fine aggregates was 2700 kg/m^3^ and 2675 kg/m^3^, respectively. A commercially available polycarboxylate-based superplasticizer (PCE) with a solid content of 50% was used to adjust the workability of concrete.

### 2.2. Mix Proportions

As shown in Table 2, thirteen concrete mix proportions were employed for the comparative study of different measurement methods for characterizing the rheological properties of the lubrication layer. Three different types of concrete, including conventional vibrated concrete (CVC), HFC, and SCC, were designed for the purpose of a comprehensive study. The mix proportion of the control sample C3 was based on a commonly used mix proportion of SCC. A water/binder ratio of 0.3 was used. The water-absorbing capacity of coarse aggregate and fine aggregate was considered. To obtain concrete in different workability, three different single variables were adopted, including the PCE dosage (C1–C5), sand ratio (C3 and C6–C9), and paste-aggregate ratio (C3 and C10–C13), which are variables often adjusted in engineering. The compressive strength of the thirteen kinds of concrete at 28d was higher than 45 MPa.

### 2.3. Testing Procedure

#### 2.3.1. Mixing

The fine aggregate and the cementitious materials were firstly added into a mixer and mixed for two minutes to ensure the homogeneity of the raw materials. Then half of the water was added to the mixer. After stirring for two minutes, the incorporation of the superplasticizer and the remaining water was accomplished, and the mixture was mixed for another two minutes. In the meantime, the wall of the mixer was manually scraped. Finally, the coarse aggregate was poured into the mixer and stirred for three minutes. Due to the high viscosity of the concrete, it took a long time to stir to make the concrete uniform. The workability of fresh concrete remained basically unchanged within one hour because of the retarding effect of the superplasticizer.

According to the mix proportions in Table 2, each sample was repeatedly prepared three times, and the following rheological measurements were performed. The average value of the three measurements was taken as the final result.

#### 2.3.2. Rheological Measurement of Constitutive Mortar

**Figure 1:**
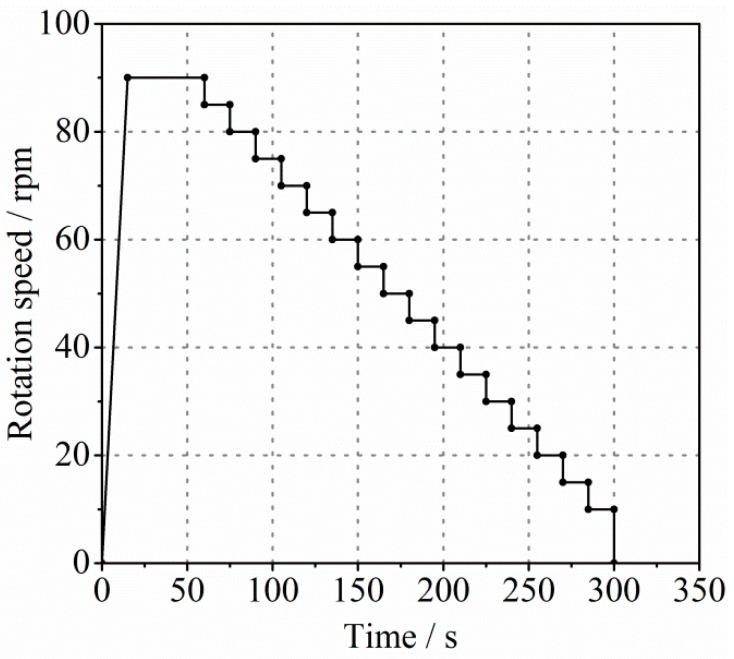
The testing scheme for measuring rheological parameters of mortar.

The constitutive mortar was obtained by screening the fresh concrete. The measurement applied was improved following the method proposed in [24,27]. A Viskomat XL coaxial rheometer, which has a four-bladed vane of the height (*h*_1_) of 70 mm and radius (*R_i_*) of 35 mm, was utilized to carry out the rheological measurement of samples. The radius (*R_o_*) of the outer cylinder was 82 mm, and the outer cylinder was ribbed to prevent the mortar from slipping. The scheme for measuring the rheological parameters of the mortar is shown in Figure 1. The total test time was 300 s, where the rotation speed remained at a maximum of 90 rpm for 45 s and then decreased step by step. This pre-shearing process minimized the impact of thixotropy before testing. In addition, high rotation speed can ensure sufficient data points without plug flow. Each platform of the testing scheme maintained a stable rotation speed for 15 s to make the mortar reach a stable flow state. Reliable experimental results can only be obtained under the condition of steady flow. The torque-rotation speed data can be acquired from the rheological measurement. The Bingham model was used as the rheology model of the mortar. Therefore, a linear relation can be obtained by fitting the torque-rotation speed data. Based on the solution of the Couette inverse problem and Reiner–Riwlin equation (Equation (1)), the rheological parameters of the mortar (the lubrication layer) can be gained through the intercept and slope of the linear relation:

(1) $$T=\frac{4\pi h_{1}\mu_{m}}{\frac{1}{R_{i}^{2}}-\frac{1}{R_{o}^{2}}}\Omega +\frac{4\pi h_{1}}{\frac{1}{R_{i}^{2}}-\frac{1}{R_{o}^{2}}}\ln\frac{R_{o}}{R_{i}}\tau_{m}$$

where *T* is the torque (N·m), Ω is the angular velocity (rad/s), $\tau_{m}$ is the yield stress of mortar (Pa), and $\mu_{m}$ is the plastic viscosity of mortar (Pa·s). The $R_{o}$ is replaced by the plug radius $R_{p}$ if the mortar is partly sheared.

#### 2.3.3. Rheological Measurement with Sliper

The rheological measurement of the lubrication layer was employed with Sliper following the method proposed in [42,43] at the same time as the mortar rheology test. Sliper needed to be pre-slid five times to form a lubrication layer on the pipe wall after the concrete was loaded into the pipe. During the test, multiple counterweights were used, and each weight was slid three times to better reflect the relationship between pressure and flow rate. The pressure sensor should be stabilized before the next slide. After each slide, the pressure and displacement were recorded automatically by Sliper. The pressure-flow rate data pairs could thus be obtained according to the pressure and displacement curves. Based on the design principle of Sliper, the measured pressure and flow rate should comply with the relationship in Equation (2):

(2) $$P=\frac{4l}{d}\tau_{s}+\frac{16\cdot l\cdot Q}{\pi \cdot d^{3}}\eta_{s}$$

(3) $$\eta_{s}=\mu_{s}/e$$

where *P* is the pressure (Pa), *Q* is the flow rate (m^3^/s), $\tau_{s}$ is the yield stress of lubrication layer (Pa), $\eta_{s}$ is the viscous constant of lubrication layer (Pa·s/m), $\mu_{s}$ is the plastic viscosity of lubrication layer (Pa·s), and *e* is the thickness of the lubrication layer (m). *l* = 0.5 m and *d* = 0.126 m were the length and diameter of the Sliper pipe, respectively. Therefore, the rheological parameters of the lubrication layer measured with Sliper could be gained according to the intercept and slope of the linear relation by fitting the pressure-flow rate data.

#### 2.3.4. Rheological Measurement of Concrete

**Figure 2:**
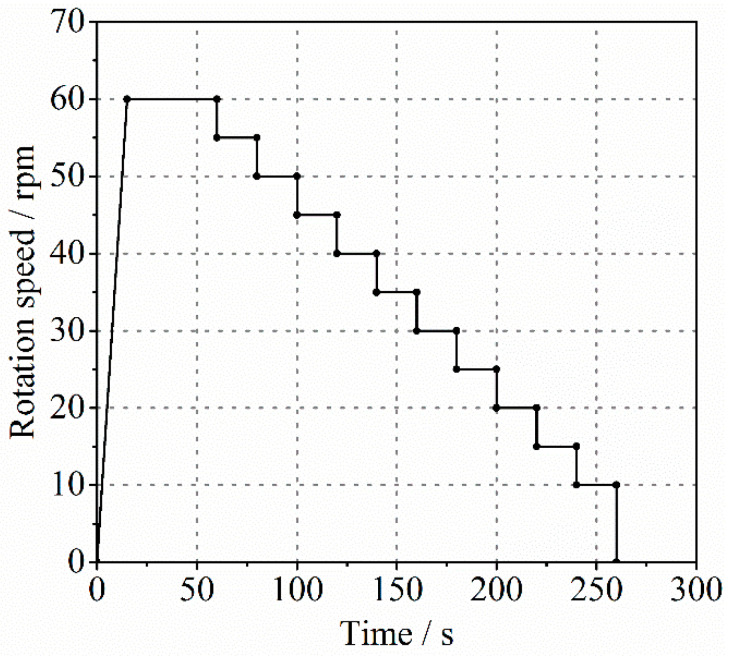
The testing scheme for measuring rheological parameters of concrete.

Since the value of concrete rheological properties is required when measuring and calculating the rheological parameters of the lubrication layer with a tribometer, the rheological measurement of fresh concrete was carried out. This also helps to understand the relationship between the rheological properties of the lubrication layer and bulk concrete. The test was started ten minutes after the concrete mixing was completed, which considered the time required to screen the concrete during the mortar test. A Viskomat XL coaxial rheometer was also utilized for this rheological measurement. The distance between the inner vane and the outer cylinder was larger than four times the size of the coarse aggregate particles. The scheme for measuring the rheological parameters of the concrete was described in Figure 2, which was similar to the testing scheme of mortar (Figure 1). As shown, the maximum rotation speed was 60 rpm, and each platform of the testing scheme maintained a stable rotation speed for 20 s. Finally, the utilization of the Reiner–Riwlin equation (Equation (1)) provided the yield stress $\tau_{c}$ and plastic viscosity $\mu_{c}$ of the fresh concrete.

Simultaneously, the slump flow and V-Funnel flow time of different concrete were determined according to the Chinese national standard GB/T 50080-2016 to investigate the variation of its workability.

#### 2.3.5. Rheological Measurement with Tribometer

The tribometer was used to carry out the rheological measurement of the lubrication layer ten minutes after the concrete mixing was finished. The test method applied was improved according to the scheme proposed in [39,40,41]. The Viskomat XL coaxial rheometer was also utilized, but the vane rotor was replaced by a steel cylinder with a radius of 35 mm. The bottom of the cylinder is conical for better insertion into concrete. The testing scheme used was the same as the mortar scheme (Figure 1). However, the pre-shearing process here was to fully form a lubrication layer over the inner cylinder wall. During actual pumping, the concrete is sheared at high speed to form a lubrication layer. The general range of the maximum shear rate in the pipe flow of pumped concrete is from 107 1/s to 653.2 1/s for the pipe with a diameter of 127 mm and 73.6 1/s to 445 1/s for the pipe with a diameter of 152 mm [39]. According to Equation (4) and assuming that the thickness of the lubrication layer was 2 mm, the maximum shear rate generated by the tribometer in this study could be approximated as 179 1/s, which is similar to the actual situation.

(4) $$\Omega ={\dot{\gamma }}_{max}\frac{R_{i}^{2}}{2}\left(\frac{1}{R_{i}^{2}}-\frac{1}{R_{l}^{2}}\right)$$

where $R_{i}$ is the radius of the inner cylinder and $R_{l}$ is the distance from the center of inner cylinder to the end of the lubrication layer in the radial direction.

In order to eliminate the bottom effect of the cone part, rheological measurements were performed at two different filling heights. The final effective height (*h*_2_) of the inner cylinder was 71 mm. The torque difference between the two experiments was the applied torque value. Since the thickness of the lubrication layer cannot be accurately measured during the test, rheological parameters of the lubrication layer were described by Equation (5):

(5) $$\tau =\tau_{t}+\eta_{t}\cdot \Omega_{l}\cdot R_{i}$$

where $\tau_{t}$ is the yield stress of lubrication layer (Pa), $\eta_{t}$ is the viscous constant of lubrication layer (Pa·s/m) and $\Omega_{l}$ is the angular velocity (rad/s), respectively. The shear stress can be transformed from the torque at the inner cylinder by Equation (6):

(6) $$\tau =\frac{T}{2\pi R_{i}^{2}h_{2}}$$

It is worth noting that the angular velocity contributed by the shear flow of the lubrication layer should be corrected based on the flowing state of the bulk concrete. That is, the effect of concrete shearing should be subtracted. The flowing state of the bulk concrete can be evaluated by the calculation of plug radius with Equation (7):

(7) $$R_{p}=\sqrt{\frac{T}{2\pi \tau_{c}h_{2}}}$$

where $\tau_{c}$ is the yield stress of concrete (Pa). If $R_{p}$ is larger than the radius of outer cylinder $R_{o}$, the bulk concrete is completely sheared. If $R_{p}$ is smaller than the radius of inner cylinder $R_{i}$, the bulk concrete is not sheared. Otherwise, the bulk concrete is partly sheared. For CVC or concrete with a high yield stress, the bulk concrete is usually not sheared. However, for HFC and SCC, which have small yield stresses or when the torque is high enough, the shear flow occurs in the bulk concrete. When the bulk concrete is completed sheared, the angular velocity contributed by the shear flow of concrete can be calculated with Equation (8):

(8) $$\Omega_{c}=\frac{T}{4\pi h_{2}\mu_{c}}\left(\frac{1}{R_{i}^{2}}-\frac{1}{R_{o}^{2}}\right)-\frac{\tau_{c}}{\mu_{c}}\ln\frac{R_{o}}{R_{i}}$$

where $\Omega_{c}$ is the angular velocity contributed by the shear flow of concrete (rad/s) and $\mu_{c}$ is the viscosity of bulk concrete. In case the bulk concrete is partly sheared, $R_{o}$ should be replaced by $R_{p}$ in Equation (8). If the bulk concrete is not sheared, $\Omega_{c}$ should be equal to zero. Moreover, $R_{i}$ should be replaced by $R_{l}$ in Equation (8) when the thickness of the lubrication layer is known.

Therefore, the $\Omega_{l}=\Omega -\Omega_{c}$ is gained for each imposed rotation speed, where Ω is the angular velocity of the cylinder. Finally, according to the corrected angular velocity and torque, the rheological parameters of the lubrication layer can be obtained with Equation (5). Furthermore, in case that the thickness of the lubrication layer has been assumed, its yield stress $\tau_{t}$ and plastic viscosity $\mu_{t}$ can be calculated directly based on the Reiner–Riwlin equation (Equation (1)) and parameters adopted here.

## 3. Results and Discussion

### 3.1. Viscosity of the Lubrication Layer

**Figure 3:**
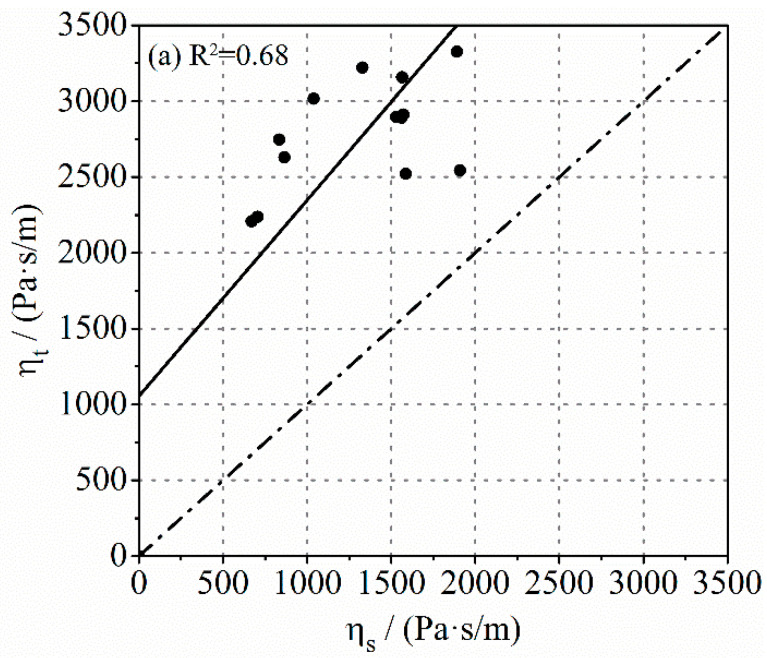

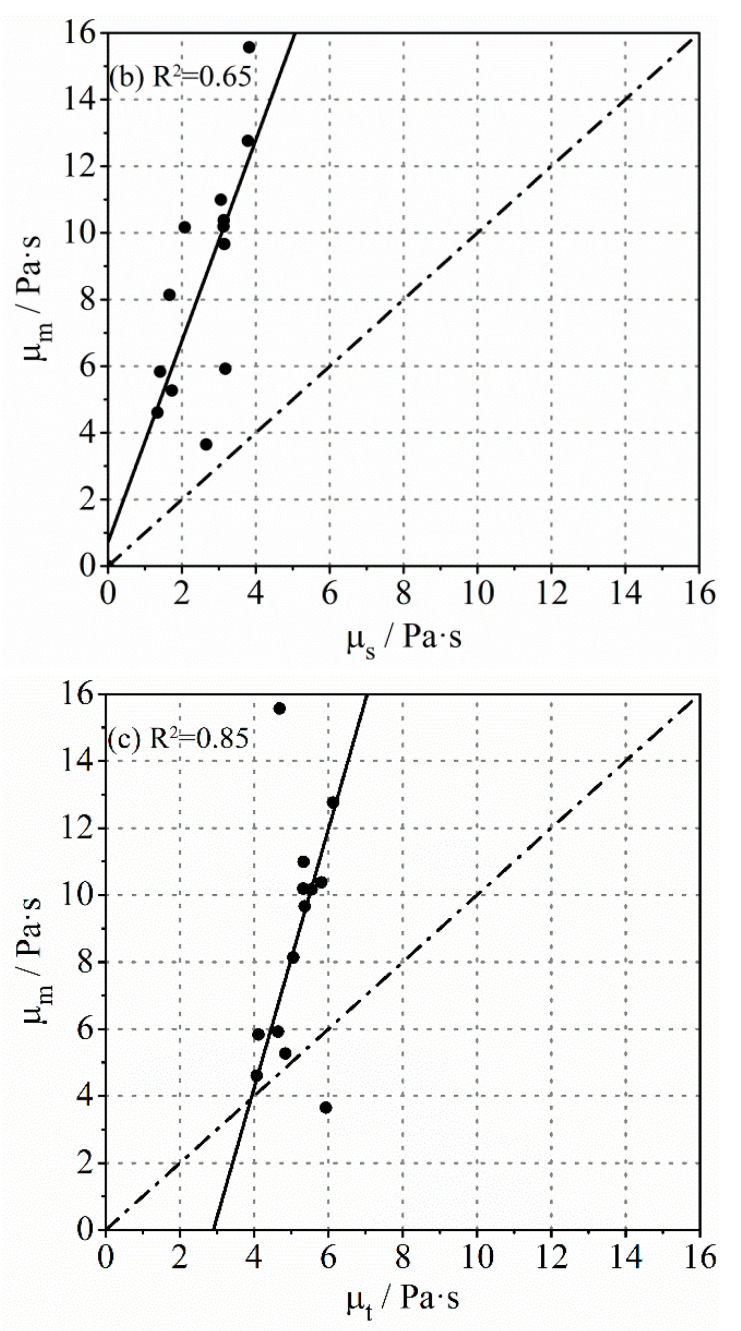
Comparison of the viscosity of the lubrication layer determined by different methods: (**a**) $\eta_{t}$ and $\eta_{s}$; (**b**) $\mu_{m}$ and $\mu_{s}$; (**c**) $\mu_{m}$ and $\mu_{t}$.

Some investigations concluded that the pressure loss during the pumping process was well correlated to the viscosity of the lubrication layer and bulk concrete, which was especially obvious for SCC and HWC [33]. Therefore, the characterization of the viscosity of the lubrication layer is vital. Figure 3 shows the relationship between the viscosities of the lubrication layer determined by different methods. The viscous constant of the lubrication layer measured by the Sliper and tribometer, respectively, is shown in Figure 3a. Theoretically, the experimental data measured from the same lubrication layer should be uniformly distributed on the dotted line or on both sides of it. However, the result measured by the tribometer is about twice higher than that measured by Sliper. On the other hand, the results obtained by both methods conform to a linear relationship to a certain extent, but the degree of linear correlation is relatively low, indicating that the data pair is somewhat scattered. In the measurement with tribometer, one of the experimental results shows a special deviation, and this data point in the figure is thus not fitted. The relationship between the plastic viscosity of mortar and the values determined by the other two methods is described in Figure 3b,c. As mentioned above, the viscosity of the lubrication layer measured with tribometer and Sliper can only be expressed in the form of viscous constant due to the unknown thickness of the lubrication layer during measurement. For a more convenient comparison, it was assumed here that the thickness of the lubrication layer formed during the test was 2 mm, which has been demonstrated in other research [28]. Its plastic viscosity could thus be calculated according to Equations (1) and (3). This did not affect the exploration of the contrast relations. It can be seen in Figure 3 that the plastic viscosity of the lubrication layer gained through the wet-screened mortar was higher than that obtained from the other two methods. This is consistent with the findings of some authors [34], who found that the measured results from the viscometer showed a 30% higher value than those of the tribometer. Moreover, the values of $\mu_{m}$ for different types of concrete are widely distributed, ranging from 4 Pa·s to 16 Pa·s. While the values of $\mu_{s}$ and $\mu_{t}$ were relatively concentrated, ranging from 1.5 Pa·s to 4 Pa·s and 4 Pa·s to 6 Pa·s, respectively. This may be due to the fact that the actual thickness of the lubrication layer was larger than 2 mm or the measuring error of the methods. Although the experimental results of different measurement systems were not similar, a good correlation between the values was observed. The linear correlation between $\mu_{m}$ and $\mu_{t}$ was more apparent.

**Figure 4:**
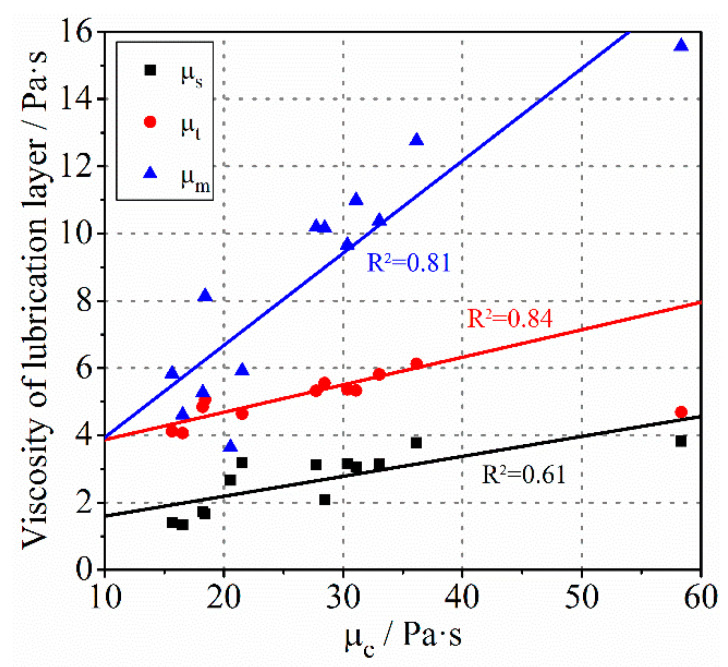
The relationship between the viscosity of concrete and the viscosity of the lubrication layer.

A rough linear relationship between the viscosity of bulk concrete and the viscous constant of lubrication layer measured with tribometer was determined by Kaplan et al., but the correlation coefficient was only 0.44 [46]. In order to study whether a similar relationship between the two exists, the plastic viscosity of the lubrication layer obtained by different methods were compared with that of the concrete. As shown in Figure 4, a good correlation was observed for different types of concrete. The correlation coefficient between $\mu_{c}$ and $\mu_{t}$ and between $\mu_{c}$ and $\mu_{m}$ both exceeded 80%, while it was not as high between $\mu_{c}$ and $\mu_{s}$. In addition, the viscosity of bulk concrete was much higher than that of the lubrication layer, which is in agreement with previous research.

### 3.2. Yield Stress of the Lubrication Layer

**Figure 5:**
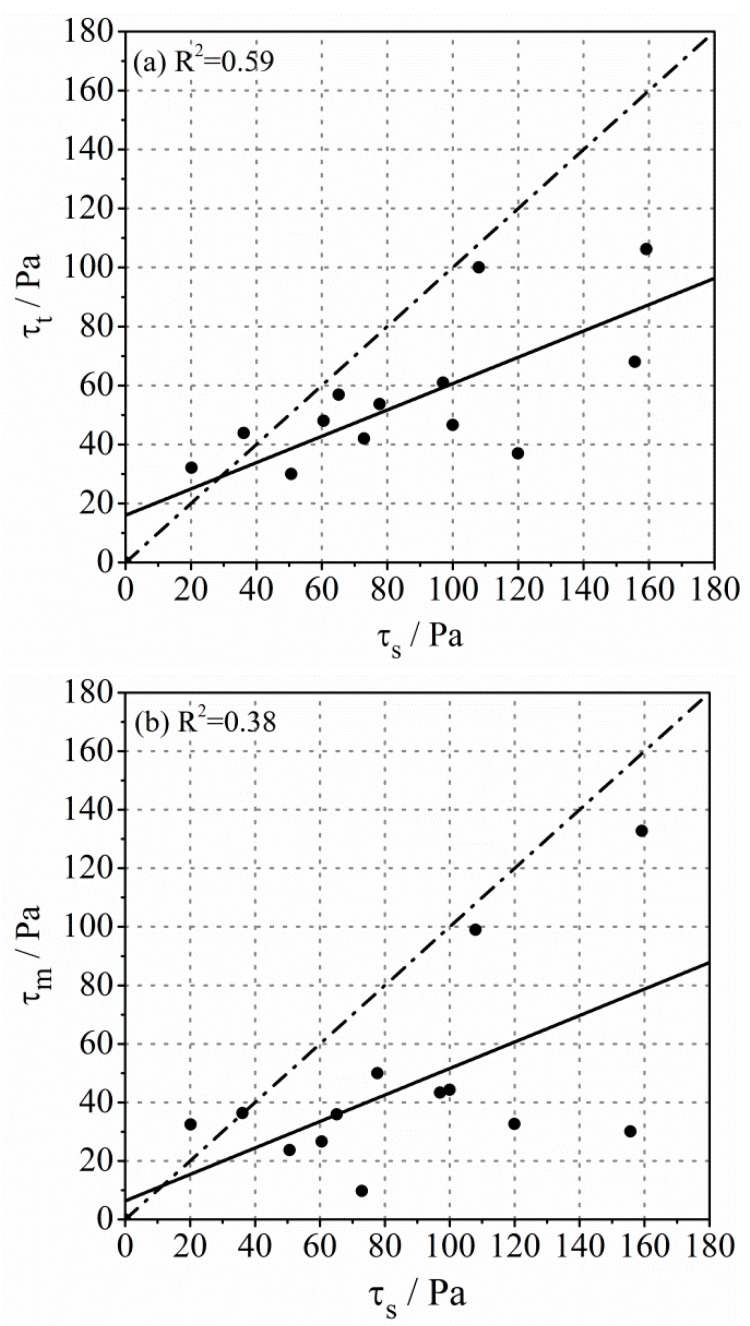

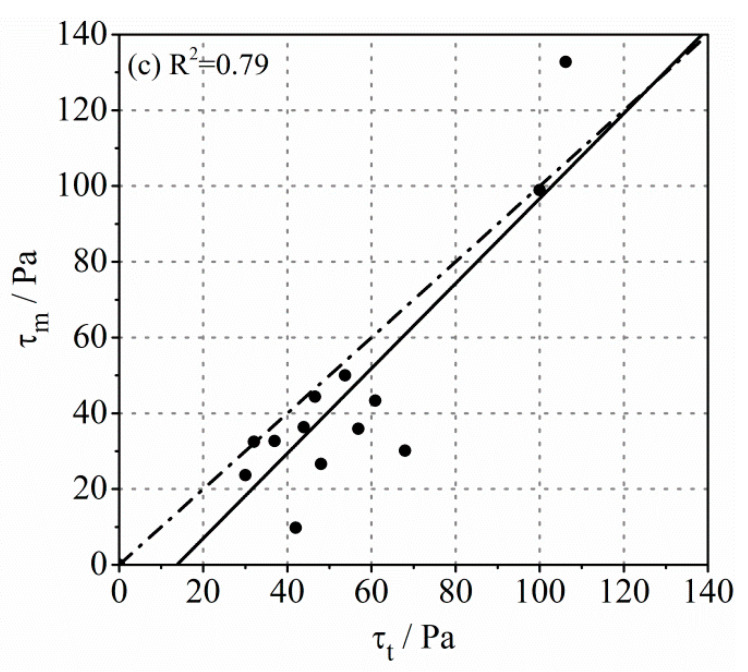
Comparison of the yield stress of the lubrication layer determined by different methods: (**a**) $\tau_{t}$ and $\tau_{s}$; (**b**) $\tau_{m}$ and $\tau_{s}$; (**c**) $\tau_{m}$ and $\tau_{t}$.

The yield stress also plays an important role in estimating the pumpability of concrete, especially at the condition of low viscosities. The yield stress of the lubrication layer investigated with different methods is displayed in Figure 5. Different from the results of viscosity, the yield stress of the lubrication layer measured by Sliper was the highest, while the yield stress of mortar was the smallest. Furthermore, the correlation between $\tau_{s}$ and $\tau_{t}$ and between $\tau_{s}$ and $\tau_{m}$ was poor, and the data points were scattered. However, the linear correlation between $\tau_{t}$ and $\tau_{m}$ was still high. Most of the data points were distributed near the dotted line, indicating that the values of $\tau_{t}$ and $\tau_{m}$ were close.

**Figure 6:**
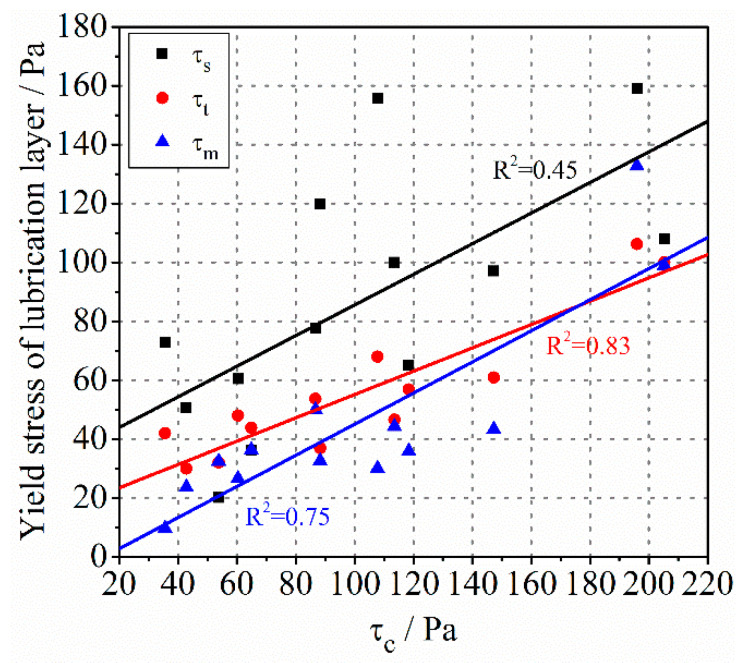
The relationship between the yield stress of concrete and the yield stress of the lubrication layer.

Some studies have shown that the yield stress of the lubrication layer was very small and even zero, which was far lower than the yield stress of bulk concrete [34]. However, some research pointed out that the yield stress of the lubrication layer may be very large and even exceed that of concrete [42]. This may depend on the composition of concrete and the measurement method. The comparison of yield stress between concrete and the lubrication layer of different types of concrete is shown in Figure 6. The yield stress of the lubrication layer measured by Sliper was in the same order of magnitude as the bulk concrete. The $\tau_{s}$ of some samples is even greater than $\tau_{c}$. It can be found that the linear correlation between them is poor. However, the $\tau_{t}$ and $\tau_{m}$ is less than $\tau_{c}$ in most cases, and the correlation coefficient has been significantly improved.

### 3.3. Workability of Concrete

**Figure 7:**
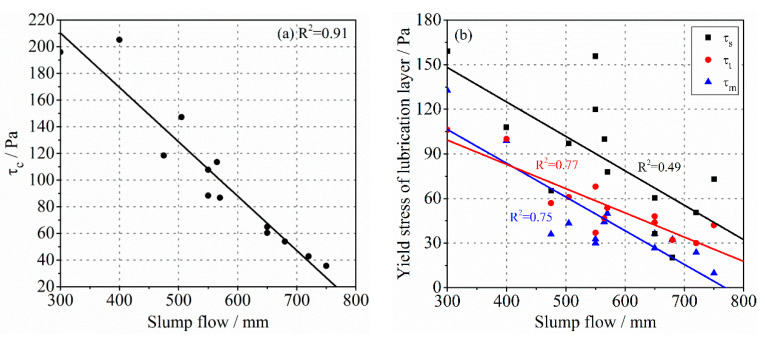
The relationship between the slump flow of concrete and the yield stress of (**a**) bulk concrete or (**b**) the lubrication layer.

**Figure 8:**
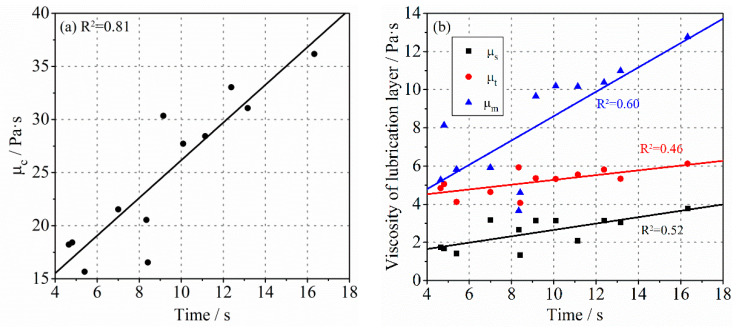
The relationship between the V-Funnel flow time of concrete and the plastic viscosity of (**a**) bulk concrete or (**b**) lubrication layer.

The workability of fresh concrete is often utilized as an index to evaluate its pumpability in engineering. The parameters commonly used to represent the workability of concrete are mainly the slump flow and V-Funnel flow time [47,48]. It is widely accepted that the slump flow and V-Funnel flow time have good correlations with the yield stress and viscosity of bulk concrete, respectively. However, whether there is a connection between them and the rheological properties of the lubrication layer has not been further explored. As shown in Figure 7, the slump flow ranges from 300 mm to 750 mm for different types of concrete, which has an obvious linear relationship with $\tau_{c}$. However, its correlation with the yield stress of the lubrication layer was weak. Especially between the slump flow and $\tau_{s}$, the data points became more scattered. The experimental results of V-Funnel flow time shown in Figure 8 are similar to that. The V-Funnel flow time has a good relationship with the plastic viscosity of the bulk concrete, while the correlation coefficient between it and the plastic viscosity of the lubrication layer is not high. It should be noted that sample C10 was blocked during the V-Funnel test, so the result was not recorded. There seemed to be no strong correlation between the workability indicators of concrete and the rheological properties of the lubrication layer. On the one hand, no lubrication layer was directly formed during the measurements of slump flow and V-Funnel flow time. On the other hand, human error could not be ignored in the process of workability measurement. For SCC, the V-Funnel flow time was only a few seconds, and the measured value deviated. These may have caused the poor correlation. Therefore, the workability of fresh concrete could represent the rheological properties of the bulk concrete to some extent. However, it is debatable to use the workability of concrete to predict the pumpability due to the decisive role of the lubrication layer in the pumping process.

### 3.4. Discussion

In general, according to the formation principle of the lubrication layer, the utilization of Sliper, tribometer, and mortar rheometer are all effective methods to determine the rheological properties of the lubrication layer. Theoretically, the calculation results should be consistent when measuring the same lubrication layer. However, as mentioned above, the experimental data gained by different methods vary significantly. The absolute values of rheological parameters, including yield stress and plastic viscosity, are different, but an obvious linear correlation between different measurement results could be observed, which indicates that there is a certain systematic deviation between different measurement systems. In fact, although the principle of the various methods was correct, the distinguishing formation processes of the lubrication layer mean that the test objects were not identical. In addition, each method had unavoidable inherent defects during the experiment, which also led to the discretization of the data points, especially when measuring with Sliper.

For Sliper, the effective length of the pipe was very short, and the sliding speed of the pipe was slow, especially when the viscosity of concrete was high. Thus, the bulk concrete is always in the state of plug flow during the measurement. The influence of the concrete shear on the formation of the lubrication layer is ignored. Moreover, it is found that the rubber ring in the middle of Sliper had a great effect on the experimental results. Friction occurs when the rubber ring is in close contact with the pipe, which prevents the pipe from sliding. Conversely, when the contact between the two is relatively loose, the mortar can leak from the gap because of its small particle size, resulting in additional friction during the slide of the pipe. It is conceivable that the friction caused by the existence of the rubber ring makes the predicted pressure-flow rate curve shift. As a result, the small viscosity and significantly large yield stress of the lubrication layer are obtained. The mix proportions of the concrete samples are diverse, and for the concrete with a large sand ratio, paste-aggregate ratio, or superplasticizer content, the phenomenon of paste leakage is more likely to occur. Therefore, the random error of each sample was different, which made the data points measured by Sliper more scattered. It was found that the lubricating oil on the rubber ring could reduce friction resistance. For the tribometer, one of the main causes of error was segregation. When the paste-aggregate ratio or the superplasticizers content was too high, the static segregation of concrete and the settlement of coarse aggregate happens. In the process of high-speed rotation of the tribometer, the concrete may also undergo dynamic segregation in the vertical direction. The segregation of concrete results in the heterogeneity of concrete. The torque at the bottom of the inner cylinder under different filling heights may thus be various, resulting in the deviation of the corrected torque. Moreover, when the mix proportion of concrete changes, the thickness of the lubrication layer may change accordingly. Therefore, the error in the assumed thickness of the lubrication layer could also cause deviation of the calculative results. Regardless, a full-scale pumping test is needed to verify the results of which test method is closer to the reality, but the results of different measures may all be useful through modification.

## 4. Conclusions

(1) The viscosity of the lubrication layer obtained through the mortar rheology was the highest, while that gained by Sliper was the smallest of all the measurement systems. However, it could be found that the yield stress determined with Sliper was significantly higher than other experimental results. The linear correlation between the rheological parameters gained by different methods was more obvious between the measurement with tribometer and mortar rheometer.
(2) There was a good linear relationship between the rheological parameters of the lubrication layer determined through tribometer or mortar rheometer and the rheological parameters of concrete. However, the correlation coefficient between the experimental results obtained with Sliper and rheological properties of concrete was not high enough.
(3) There was a good linear relationship between the workability indicators and the rheological parameters of bulk concrete. The workability of fresh concrete might only be suitable to predict the rheological properties of bulk concrete.
(4) The friction caused by the rubber ring and the leaking mortar during the Sliper measuring results in a significantly overestimated yield stress of the lubrication layer. The segregation of concrete leads to the error of experimental results obtained by the tribometer. The difference in the formation of the lubrication layer results in the systematic deviation between different methods.

## Figures and Tables

**Table 1:** Chemical compositions of cement, fly ash and silica fume *w*/%.

Composition	CaO	SiO_2_	Al_2_O_3_	Fe_2_O_3_	MgO	SO_3_	Na_2_O_eq_
Cement	63.27	22.59	4.42	3.44	2.43	2.41	0.38
Fly ash	4.59	48.98	31.43	9.13	0.49	1.52	1.06
Silica fume	0.26	97.12	0.06	0.18	0.36	0.1	1.21

Note: Na_2_O_eq_ = Na_2_O + 0.658 K_2_O.

**Table 2:** Mix proportions of concrete (kg/m^3^).

Sample	Cement	Fly Ash	Silica Fume	Fine Aggregates	Coarse Aggregates	PCE	Sand Ratio/%	Paste-Aggregate Ratio/%	W/B Ratio
C1	401	150	25	823	794	4.90	51	63	0.3
C2	401	150	25	823	794	5.48	51	63	0.3
C3	401	150	25	823	794	6.34	51	63	0.3
C4	401	150	25	823	794	7.20	51	63	0.3
C5	401	150	25	823	794	8.35	51	63	0.3
C6	401	150	25	728	890	6.34	45	63	0.3
C7	401	150	25	776	841	6.34	48	63	0.3
C8	401	150	25	873	744	6.34	54	63	0.3
C9	401	150	25	922	695	6.34	57	63	0.3
C10	319	119	21	933	900	6.34	51	44	0.3
C11	360	135	23	878	847	6.34	51	53	0.3
C12	442	165	29	768	741	6.34	51	75	0.3
C13	483	181	31	714	688	6.34	51	88	0.3

## Data Availability

The data presented in this study are available in the article.

## List of Symbols

$h_{1}$	height of vane rotor (m)
$R_{i}$	radius of the vane rotor or the inner cylinder (m)
$R_{o}$	radius of the outer cylinder (m)
T	torque (N·m)
Ω	angular velocity of cylinder (rad/s)
$\tau_{m}$, $\tau_{s}$, $\tau_{t}$	yield stress of lubrication layer measured by mortar rheometer, Sliper, and tribometer, respectively (Pa)
$\mu_{m}$, $\mu_{s}$, $\mu_{t}$	plastic viscosity of lubrication layer measured by mortar rheometer, Sliper, and tribometer, respectively (Pa·s)
$R_{p}$	radius of cross-section of the plug flow (m)
P	pressure (Pa)
Q	flow rate (m^3^/s)
$\eta_{s}$, $\eta_{t}$	viscous constant of the lubrication layer measured using Sliper and tribometer, respectively (Pa·s/m)
e	thickness of lubrication layer (m)
l	effective length of the Sliper pipe (m)
d	diameter of Sliper pipe (m)
$R_{l}$	distance from the center of the inner cylinder to the end of the lubrication layer in a radial direction (m)
$\tau_{c}$	yield stress of concrete (Pa)
$\mu_{c}$	plastic viscosity of concrete (Pa·s)
$\dot{\gamma }$	shear rate (s^−1^)
$\Omega_{l}$	angular velocity contributed by the shear flow of lubrication layer (rad/s)
$h_{2}$	effective height of the inner cylinder (m)
τ	shear stress (Pa)
$\Omega_{c}$	angular velocity contributed by the shear flow of concrete (rad/s)
